# Third-variable effect analysis with multilevel additive models

**DOI:** 10.1371/journal.pone.0241072

**Published:** 2020-10-23

**Authors:** Qingzhao Yu, Bin Li

**Affiliations:** 1 Biostatistics Program, School of Public Health, Louisiana State University Health Science Center, New Orleans, LA, United States of America; 2 Department of Experimental Statistics, Louisiana State University, Baton Rouge, LA, United States of America; Texas A&M University, UNITED STATES

## Abstract

Third-variable effect refers to the effect transmitted by third-variables that intervene in the relationship between an exposure and a response variable. Third-variable effect analysis has been broadly studied in many fields. However, it remains a challenge for researchers to differentiate indirect effect of individual factor from multiple third-variables, especially when the involving variables are of hierarchical structure. Yu et al. (2014) defined third-variable effects that were consistent for all different types of response (categorical or continuous), exposure, or third-variables. With these definitions, multiple third-variables can be considered simultaneously, and the indirect effects carried by individual third-variables can be separated from the total effect. In this paper, we extend the definitions of third-variable effects to multilevel data structures, where multilevel additive models are adapted to model the variable relationships. And then third-variable effects can be estimated at different levels. Moreover, transformations on variables are allowed to present nonlinear relationships among variables. We compile an R package *mlma*, to carry out the proposed multilevel third-variable analysis. Simulations show that the proposed method can effectively differentiate and estimate third-variable effects from different levels. Further, we implement the method to explore the racial disparity in body mass index accounting for both environmental and individual level risk factors.

## Introduction

A third-variable effect (TVE) refers to the effect conveyed by a third-variable to an observed relationship between an exposure and a response variable of interest. Depend on whether there is a causal relationship from the exposure variable to the third-third variable and then to the response, the third-variable (denoted as *M*) is often called a mediator (when there are causal relationships) or a confounder (no causal relationship is involved). Research purposes for the third-variable analysis are generally, 1) identify significant third-variables that can partially or completely explain the relationship between the exposure variable (*X*) and the outcome (*Y*); and 2) differentiate the TVE from different paths that connect between *X* and *Y*. Except for the difference in explaining the effects from a mediator and from a confounder, the TVE analysis method can be used to make inferences on both mediation and confounding effects [1]. Third-variable analysis has been widely used in psychology, social sciences, behavioral research, health prevention, epidemiological studies, and genetic analysis. Within the context of generalized linear models, general predictive models, and counterfactual framework, a number of methods have been proposed for inferences on TVEs, to name a few, [2–7].

In research practices, the experiment data are often collected hierarchically. For example, to identify variables that are related with childhood obesity, we consider both environmental (e.g. walkability of the neighborhood) and individual factors (e.g. snacking habit). When hierarchical databases are considered, third-variable analysis method based on generalized linear models are usually not readily adaptable since the independence assumption among observations is violated. In such cases, hierarchical models, also known as multilevel or mixed-effect models, are more appropriate to fit relationships among variables since these models can catch dependencies among observations and allow for predictors from different levels of the data [8]. In this paper, we propose a third-variable analysis method based on multilevel models. For the hierarchical model, we assume there are two levels of data and refer the individual level as level 1, and the group level as level 2. Although more than two levels of hierarchy is possible, this paper focuses on two-level databases only.

In third-variable analysis, besides the pathway that directly connect the exposure variable with the outcome, we explore the *exposure* → *third*-*variable* → *response* or *X* → *M* → *Y* pathways. When doing third-variable analysis with multilevel models, the level of the variables at the left of the arrow should be higher than or equal to that of the right, since a group level variable may affect an individual level variable but not vice versa [9]. In this setting, only the 2 → 2 → 2, 2 → 2 → 1, 2 → 1 → 1, and 1 → 1 → 1 relationships are legible. Multilevel models are necessary to deal with hierarchical data base even for the 1 → 1 → 1 relationship. [10] studied the bias brought by using single level models when data are hierarchical. [9, 11–13] proposed third-variable analysis methods for all three types of multilevel models. Moreover, [14–16] proposed alternative methods to test the indirect effects in 2 → 2 → 1, 2 → 1 → 1 models. In addition, [17] proposed to use Bayesian third-variable analysis to deal with hierarchical databases.

In this paper, we propose to use generalized additive multilevel models for third-variable analysis with hierarchical databases. The major contributions of this proposed method are that: 1) we extend the general definitions of TVEs proposed by [7] to multilevel data structure; 2) the method allows any types (categorical or continuous) of the exposure(s), third-variables and response variable(s) in exploring TVEs; 3) TVEs from individual or group of third-variables and from different levels are differentiable; 4) nonlinear associations among variables are allowed in calculating the TVEs; 5) multiple exposure(s) from different levels and multivariate outcomes are allowed in the third-variable analysis, and 6) an R package *mlma* was developed for the method proposed in this paper. Practitioners can easily implement the method in real data analysis.

The rest of the paper is organized as follows. In Section 2, we review the general definitions of TVEs and then extend these concepts to the multilevel model situation. We also review the generalized additive multilevel models (GAMM) that are used to model relationships among variables. Based on that, we propose the multilevel third-variable analysis with GAMM. In Section 3, we illustrate the use of the proposed method in different multilevel data structures and the usage of the *mlma* R package. We then use the method in a real data example in Section 4: to explore the ethnic disparity in obesity considering both individual and environmental risk factors. Section 5 gives a summary of the proposed method and points out future research directions.

## 2 Third-variable analysis with multilevel additive models

[7] proposed general definitions for TVEs. First we recapitulate the definitions and then propose the multilevel additive models in third-variable analysis with hierarchical databases.

### 2.1 Definitions of third-variable effects with single-level data

**Fig 1:**
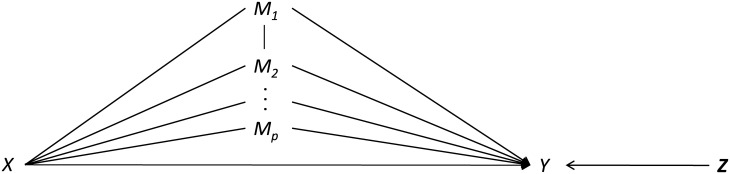
Conceptual model for one-level third-variable effects.

The conceptual model for TVE of one level is shown in Fig 1. In the Figure, *X* is the exposure variable, *Y* is the outcome, **M** = {*M*_1_, …, *M*_*p*_} is the vector of *p* third-variables, and **Z** is the vector of other variables which relate with *Y* but do not intermediate the *X*−*Y* relationship. As usual, the TVE includes total effect—the overall effect from the exposure variable *X* to the outcome *Y*, direct effect—the remaining effect from *X* to *Y* after accounting for effects form third-variables, and indirect effect from *M*_*i*_—the effect from the path *X*−*M*_*i*_−*Y*.

Basically, [7] defines the total effect as the *changing rate* in the outcome when the exposure variable changes. Compared with the traditional definition of TVEs, which are defined as the *amount of change* of the outcome when the exposure variable changes from one status to another, the Yu et al.’s definition has the following benefits:

1. The total effect is scale invariant to the unit of the exposure variable. This is because the effect is defined as the changing rate but not as the changing amount of *Y* with *X*.
2. There is no need to define the two status of the exposure variable. Therefore the total effects can be calculated not only for binary but also for continuous exposures.
3. The total effect can be calculated with nonlinear models when the relationship among variables are changing at different values of the exposure variable. The total effect can be expressed as a function of the exposure variable by the Yu definition [7].

The direct effect not from *M*_*i*_ is similarly defined as the total effect except that the relationship between the exposure variable and *M*_*i*_ is manipulatively broken. In such case, *M*_*i*_ are controlled not to change with *X*: *M*_*i*_ follows its marginal distribution from the observations. The indirect effect from *M*_*i*_ is then defined as the total effect minus the direct effect not from *M*_*i*_. All the benefits of the definition of total effect are also fit for the direct and indirect effects. In additon, multiple third-variables can be considered simultaneously, and the indirect effects carried by individual third-variables can be separated from the total effect. For the formal definitions of TVEs, readers are referred to [7] and [18]. [7] showed that the proposed definitions of TVEs are equivalent to the conventional TVEs under certain situations (e.g., linear regression models with continuous third-variables and outcome). They also established the relationship between the proposed definitions of direct or indirect effect and the natural direct or indirect effect for binary exposure variables. Later, the definitions have been implemented to explore racial and ethnic health disparities by [19, 20] and extended to deal with time-to-event outcomes ([21]) and for multiple exposures and multivariate outcomes ([22]). In this paper, we extend the method to the context of multilevel models.

### 2.2 Definitions of third-variable effects with data of two levels

The unique data structure in multilevel models raises the potential problem of confounding TVEs from different levels. As pointed out in [13], the relationship between two level-one variables can be decomposed into between-group and within-group components. In particular, the aggregated variables at the second level can be highly related while the relationship may be very weak or even at an opposite direction when considered at the individual level [23]. For example, [24] points out that the proportion of black residents may be an important variable for the census tract, while it is different from the meaning of ethnicity as an individual-level variable. [25] discussed the difference of the two components extensively. It is important to differentiate the between-group and within-group components in third-variable analysis, where the TVEs can be decomposed to level 1 and level 2 effects. To identify the level 1 and level 2 TVEs separately, [13] proposed the group-mean centering method (CWC), where they subtracted the group means from individual level variables and added group means as level 2 covariates. In their paper, [13] showed that the CWC method efficiently separated level 1 and level 2 TVEs and resulted in less bias and more power compared with non-centering methods. In this paper, we use a different way to estimate the level 1 and level 2 TVEs: extend the definitions of TVEs with single level models by [7] to multilevel models.

With the generalized definition of TVEs, [22] has shown that a third-varaible analysis can involve multiple exposure variables and multivariate outcomes. The purpose of third-variable analysis is to differentiate the direct effect and indirect effect from each third-variable for each pair of the exposure-outcome relationship. If the outcome is at level 2, all exposure and mediators have to be level 2 as discussed in Section 1. Therefore, a single-level third-variable analysis works. If the outcome is at level 1, the exposure variable can be a level 1 or level 2 variable. The third-variables can be level 1 or 2 for a level 2 exposure, but have to be level 1 for a level 1 exposure variable. In this paper, we focus on level 1 outcomes.

Denote **M**_*ij*_ = (*M*_*ij*1_, …, *M*_*ijK*_) as the vector of *K* potential level 1 third-variables for the *ith* object at the *jth* group. **M**_*ij*,−*k*_ is the vector **M**_*ij*_ excluding the *kth* element. Denote **M**_.*j*_ = (*M*_.*j*1_, …, *M*_.*jL*_) as the vector of the *L* potential level 2 third-variables or level 1 third-variables aggregated at level 2 within group *j*. Let *M*_*ijk*_(*x*_*ij*_) be a random variable that has a conditional distribution given *X*_*ij*_ = *x*_*ij*_. For an exposure variable *X* at any level, let u* be the minimum unit of *X*, such that if *x* ∈ *domain*(*X*), then *x* + u* ∈ *domain*(*X*). For now, we ignore other covariates **Z**. Assume effects of exposures and third-variables on the outcome are additive, we have the general definitions of TVEs, following [7], for level 1 (Definition 1) and level 2 exposure variables (Definition 2). Note that a level 1 exposure can have only level 1 mediators while a level 2 exposure can have both level 1 and level 2 mediators.

**Definition 1**. *For a level 1 exposure variable X, the level 1 total effect (TE*_1_) *of X on Y, the level 1 direct effect* ($DE_{1,\backslash M_{k}}$) *of X on Y not from level 1 third-variable M*_*k*_ *and the level 1 indirect effect of X on Y through M*_*k*_ *at X* = *x*_*ij*_ ($IE_{1,M_{k}}$) *are defined as*:

$$\begin{array}{ccc} TE_{1}\left(x_{ij}\right) & = & \lim_{u\to u*}\frac{EY_{ij}\left(x_{ij}+u,M_{ij}\left(x_{ij}+u\right),x_{.j},M_{.j}\right)-EY_{ij}\left(x_{ij},M_{ij}\left(x_{ij}\right),x_{.j},M_{.j}\right)}{u}; \end{array}$$ (1)

$$\begin{array}{ccc} DE_{1,\backslash k}\left(x_{ij}\right) & = & \lim_{u\to u*}E_{M_{ijk}}[\frac{EY_{ij}\left(x_{ij}+u,M_{ij,-k}\left(x_{ij}+u\right),M_{ijk},x_{.j},M_{.j}\right)}{u};\\ & & \;\;\;\;\;\;\;\;\;\;\;\;\;\;\;\;-\frac{EY_{ij}\left(x_{ij},M_{ij,-k}\left(x_{ij}\right),M_{ijk},x_{.j},M_{.j}\right)}{u}] \end{array}$$ (2)

$$\begin{array}{ccc} IE_{1,k}\left(x_{ij}\right) & = & TE_{1}\left(x_{ij}\right)-DE_{1,\backslash k}\left(x_{ij}\right). \end{array}$$ (3)

The average level one TVEs are the mean value of the TVEs defined by Definition 1: *ATE*_1_ = *E*_*ij*_[*TE*_1_(*x*_*ij*_)], *ADE*_1,\*k*_ = *E*_*ij*_[*DE*_1,\*k*_(*x*_*ij*_)] and *AIE*_1,*k*_ = *ATE*_1_ − *ADE*_1,\*k*_.

**Definition 2**. *For a level 2 exposure variable X, the level 2 total effect* (*TE*_2_) *of X on Y, the level 2 direct effect* ($DE_{2,\backslash M_{k}}$) *of X on Y not from the level 1 third variable M*_*k*_ *and level 2 third variable M*_*l*_, *and the level 2 indirect effect of X on Y through M*_*k*_ *and M*_*l*_ *at X* = *x*_.*j*_ ($IE_{1,M_{k}}$) *are defined as*:

$$\begin{array}{ccc} TE_{2}\left(x_{.j}\right) & = & \lim_{u\to u*}E_{i}[\frac{EY_{ij}\left(x_{ij},M_{ij}\left(x_{.j}+u\right),x_{.j}+u,M_{.j}\left(x_{.j}+u\right)\right)-EY_{ij}\left(x_{ij},M_{ij}\left(x_{.j}\right),x_{.j},M_{.j}\left(x_{.j}\right)\right)}{u}]; \end{array}$$ (4)

$$\begin{array}{ccc} DE_{21,\backslash k}\left(x_{.j}\right) & = & \lim_{u\to u*}E_{i}E_{M_{ijk}}[\frac{EY_{ij}\left(x_{ij},M_{ij,-k}\left(x_{.j}+u\right),M_{ijk},x_{.j}+u,M_{.j}\left(x_{.j}+u\right)\right)}{u};\\ & & \;\;\;\;\;\;\;\;\;\;\;\;\;\;\;\;\;\;-\frac{EY_{ij}\left(x_{ij},M_{ij,-k}\left(x_{.j}\right),M_{ijk},x_{.j},M_{.j}\left(x_{.j}\right)\right)}{u}]; \end{array}$$ (5)

$$\begin{array}{ccc} DE_{22,\backslash l}\left(x_{.j}\right) & = & \lim_{u\to u*}E_{i}E_{M_{.jl}}[\frac{EY_{ij}\left(x_{ij},M_{ij}\left(x_{.j}+u\right),x_{.j}+u,M_{.j,-l}\left(x_{.j}+u\right),M_{.jl}\right)}{u};\\ & & \;\;\;\;\;\;\;\;\;\;\;\;\;\;\;\;\;\;-\frac{EY_{ij}\left(x_{ij},M_{ij}\left(x_{.j}\right),x_{.j},M_{.j,-l}\left(x_{.j}\right),M_{.jl}\right)}{u}]; \end{array}$$ (6)

$$\begin{array}{ccc} IE_{21,k}\left(x_{.j}\right) & = & TE_{2}\left(x_{.j}\right)-DE_{21,\backslash k}\left(x_{.j}\right); \end{array}$$ (7)

$$\begin{array}{ccc} IE_{22,l}\left(x_{.j}\right) & = & TE_{2}\left(x_{.j}\right)-DE_{22,\backslash l}\left(x_{.j}\right). \end{array}$$ (8)

The average level 2 TVEs are the TVEs defined by Definition 2 averaged at the group level: *ATE*_2_ = *E*_*j*_[*TE*_2,*j*_(*x*_.*j*_)], *AIE*_21,*k*_ = *E*_*j*_[*IE*_21,*jk*_(*x*_.*j*_)] and *AIE*_22,*l*_ = *E*_*j*_[*IE*_22,*jl*_(*x*_.*j*_)].

### 2.3 Multilevel additive models

We use multilevel additive models to build relationships among variables of hierarchy. The additive model is a nonlinear regression method that was first proposed by [26]. A multilevel additive model can deal with both nonlinear covariate effects and cluster-specific heterogeneity [27]. It is now gaining rapid popularity in psychological and social research [28]. Using the notations in Section 2.2, assume that we have *L* level 2 and *K* level 1 third-variables. In addition, assume that there are *E*_1_ level 1 and *E*_2_ level 2 exposure variables, denoted as $X_{ij}^{T}=\left\{X_{ij1},\ldots ,X_{ijE_{1}}\right\}$ and $X_{.j}^{T}=\left\{X_{.j1},\ldots ,X_{.jE_{2}}\right\}$ respectively. We propose the following linear additive multilevel models for multilevel third-variable analysis. The boldfaced letter indicates a potential vector of functions or numbers.

For level 2 third-variables, *M*_.*jl*_, *l* = 1, …, *L*:

$$\begin{array}{c} g_{.l}\left(E\left(M_{.jl}\right)\right)=\alpha_{0l}+\sum_{e=1}^{E_{2}}{\alpha_{2le}}^{T}f_{2le}\left(X_{.je}\right). \end{array}$$

For level 1 third-variables, *M*_*ijk*_, *k* = 1, …, *K*:

$$\begin{array}{ccc} g_{1k}\left(E\left(M_{ijk}\right)\right) & = & u_{0jk}+\sum_{e=1}^{E_{1}}{\alpha_{1ke1}}^{T}f_{1ke1}\left(X_{ije}\right)+\sum_{e=1}^{E_{2}}{\alpha_{2ke1}}^{T}f_{2ke1}\left(X_{.je}\right);\\ u_{0jk} & = & \alpha_{00k}+r_{0jk}. \end{array}$$

The full model:

$$\begin{array}{ccc} E(Y_{ij}) & = & u_{0j}+\sum_{e=1}^{E_{1}}{\beta_{1e}}^{T}f_{1e}\left(X_{ije}\right)+\sum_{e=1}^{E_{2}}{\beta_{2e}}^{T}f_{2e}\left(X_{.je}\right)+\sum_{k=1}^{K}{\beta_{3k}}^{T}f_{3k}\left(M_{ijk}\right)+\sum_{l=1}^{L}{\beta_{4l}}^{T}f_{4l}\left(M_{.jl}\right);\\ u_{0j} & = & \beta_{00}+r_{0j}. \end{array}$$

In the models, *r*_0*jk*_ and *r*_0*j*_ are second-level random errors with mean zero and finite variances. **f**(⋅) is a function/transformation vector of ⋅. The transformation enables modeling nonlinear relationships among variables. We assume that all the transformation functions are first-order differentiable. *α* and *β* are coefficient vectors for transformed variables in predicting the response variable on the left side of each equation. In addition, *g*(⋅)s are the link functions that link the expected response variable with the right-hand-side of each equation, the systematic component of a generalized linear model. For example, a binary *M*_*ijk*_ may have a link function *g*_1*k*_ = *logit*(*P*(*M*_*ijk*_ = 1)). Similarly, a link function can be used on the outcome. With the link function, we can deal with different types of third-variables and outcomes.

### 2.4 Third-variable effects with multilevel additive model

Based on the definitions of TVEs, we derive the TVEs in Theorems 1 and 2. In the theorems, *f*′(*x*) denotes the first derivative of function *f* on the random variable *X* and realized at *X* = *x*. In addition, *g*^−1^ denotes the inverse function of *g*. We further denote *μ*_*ijk*_ = *E*(*M*_*ijk*_) and *μ*_.*jk*_ = *E*(*M*_.*jk*_). The proofs of theorems are included in the S1 File.

**Theorem 1** *With the relationships among variables built by Section 2.3, the TVEs for level 1 exposure variable X*_*ije*_, *e* = 1, …, *E*1 *on the outcome variable Y are*:

$$\begin{array}{ccc} IE_{1,k}\left(x_{ije}\right) & = & \left[{\alpha_{1ke1}}^{T}f_{1ke1}^{\prime }\left(x_{ije}\right)\cdot g_{1k}^{-1\prime }\left(\mu_{ijk}\right)\right]\times \left[{\beta_{3k}}^{T}{f_{3k}}^{\prime }\left(m_{ijk}\right)\right],k=1,\ldots K\\ DE_{1}\left(x_{ije}\right) & = & {\beta_{1e}}^{T}{f_{1e}}^{\prime }\left(x_{ije}\right)\\ TE_{1}\left(x_{ije}\right) & = & DE_{1}\left(x_{ije}\right)+\sum_{k=1}^{K}IE_{1,k}\left(x_{ije}\right) \end{array}$$

**Theorem 2**. *With the relationships among variables built by Section 2.3, the TVEs for level 2 exposure variable X*_.*je*_, *e* = 1, …, *E*2 *on the outcome variable Y are*:

$$\begin{array}{ccc} IE_{22,l}\left(x_{.je}\right) & = & \left[{\alpha_{2le}}^{T}f_{2le}^{\prime }\left(x_{.je}\right)\cdot g_{.l}^{-1\prime }\left(\mu_{.jl}\right)\right]\times \left[{\beta_{4l}}^{T}{f_{4l}}^{\prime }\left(m_{.jl}\right)\right],l=1,\ldots L\\ IE_{21,k}\left(x_{.je}\right) & = & \left[{\alpha_{2ke1}}^{T}f_{2ke1}^{\prime }\left(x_{.je}\right)\cdot g_{1k}^{-1\prime }\left(\mu_{ijk}\right)\right]\times \left[{\beta_{3k}}^{T}E{f_{3k}}^{\prime }\left(m_{ijk}\right)\right],k=1,\ldots K\\ DE_{2}\left(x_{.je}\right) & = & {\beta_{2e}}^{T}{f_{2e}}^{\prime }\left(x_{.je}\right)\\ TE_{1}\left(x_{ije}\right) & = & DE_{2}\left(x_{.je}\right)+\sum_{k=1}^{K}IE_{21,k}\left(x_{.je}\right)+\sum_{l=1}^{L}IE_{22,l}\left(x_{.je}\right) \end{array}$$

### 2.5 Bootstrap method for third-variable effect inferences

Finally, we use the bootstrap method to calculate the variances of the TVEs. In particular, at the group level, a bootstrap sample of the same size for each group is drawn with replacement from the original data set. Then multilevel additive models are fitted based on the bootstrap sample to get the estimates of *β*s, based on which the TVEs can be calculated by Theorems 1 and 2. The above process repeats many times and inferences can be made based on the repeated estimates. To draw the bootstrap sample, we keep all groups at the second level and then at the individual level, we draw observations with replacement of size *n*_*j*_ from the jth group, where *n*_*j*_ is the number of observations in the jth group. This bootstrap method is adopted in the R package *mlma* to estimate the variances of estimates and to build up confidence intervals. The *mlma* package is available from the Comprehensive R Archive Network (CRAN) at https://cran.r-project.org/web/packages/mlma/index.html and illustrated by the simulations in Section 3.

### 2.6 The *mlma* R package

The authors developed a R package, *mlma*, for multilevel third-variable analysis. The analysis is based on multilevel additive models where nonlinear transformations of variables are allowed. The package **mlma** contains three groups of functions: function *data.org* is used to prepare data sets for analysis—transforming variables, dichotomizing categorical third-variables, and getting the derivatives of the transformation functions. The functions *mlma* and *boot.mlma* are used for statistical inferences on the TVEs. The former estimates the TVEs and the latter generates bootstrap samples from the original data sets and does the third-variable analysis based on the bootstrap samples. The estimates of TVEs from the bootstrap samples are then used for statistical inferences. The third group of functions are generic functions—*print, summary* and *plot* results from the *mlma* and *boot.mlma* functions. The details of how to use the functions are fully documented within the package and the mlma vignette. The analysis in Section 3 are all performed using the package.

## 3 Simulations

In this section, we show the performance of the proposed method and illustrate the use of the R package *mlma*. The first simulation is based on a 2 → 1 → 1 true model adapted from [13] but it is extended to include exposure and third-variables at both levels. There is one exposure variable at each level and one third-variable at each level. The level 1 exposure and level 2 third-variable are binary. No transformation is performed on the original variables. The first simulation is to exam how the sensitivity and specificity of identifying important third-variables are influenced by different sets of parameters.

The second simulation is based on nonlinear multilevel models with both level exposures and one level 1 continuous third-variable. The second simulation is to demonstrate how to include transformation terms of variables in the multilevel mediation analysis and how to use the graph tools in the R package *mlma* to illustrate relationships among variables.

The simulations are performed using the R package *mlma*. The R codes are included in the S1 File.

### 3.1 Simulation 1

The first simulation includes one level 1 binary exposure, *X*_*ij*_, and one level 2 continuous exposure, *X*_.*j*_, where *j* denotes the *jth* group and *i* denotes the ith observation in the *jth* group. There are 600 observations in total and *n* observations in each group. Therefore, there are 600/*n* groups. To check how group sizes and number of groups can influence the sensitivity of identifying important TVEs, we set *n* at 5 and 20 respectively. *X*_*ij*_ are generated through binomial distribution with the probability of success 0.5. *X*_.*j*_ are generated through a standard normal distribution. There are a level 2 binary third variable, *M*_.*j*_, and one level 1 continuous third-variable, *M*_*ij*_. In detail, the simulation data are generated from the following models for *i* = 1, …, *n* and *j* = 1, …, 600/*n*:

$$\begin{array}{ccc} logit(m_{.j}=1) & = & 0.8x_{.j},\\ m_{ij} & = & u_{0j}+0.8x_{.j}+0.8x_{ij}+\epsilon_{0ij},\\ y_{ij} & = & u_{1j}+\beta_{1}x_{ij}+\beta_{2}x_{.j}+\beta_{3}m_{ij}+\beta_{4}m.j+\epsilon_{1ij}, \end{array}$$

where *u*_0*j*_ ∼ *N*(0, 0.5), *ϵ*_0*ij*_ ∼ *N*(0, 1), *u*_1*j*_ ∼ *N*(0, *v*_2_) and *ϵ*_1*ij*_ ∼ *N*(0, *v*_1_) are independently generated random errors at both levels. The level two random error for the response variable is set as one-fifth of the level one variance—*v*_2_ = *v*_1_/5, which makes the intraclass correlation (ICC) to be.17. As pointed out by [29], this medium ICC value facilitates model convergence. *v*_2_ is chosen to be 5 or 1, *β*_1_ and *β*_2_ ranges within the set {−.59, −.14, 0, 0.14, 0.39} and *β*_3_ and *β*_4_ from {0, 0.14, 0.59}. We then check the influence of these parameters on the sensitivity and specificity of identifying important TVEs. The data with each combination of parameters are generated 20 times. The sensitivity of a TVE is the proportion of times that the estimated confidence interval of the effect does not include 0 when the actual effect is not 0. Relatively, the specificity is defined as the proportion of times that the estimated confidence interval of the effect includes 0 when the true effect is 0.

For the level 2 exposure, the average direct effect for *X*_.*j*_ → *Y*_*ij*_ (denoted as de2), the indirect effect from *M*_.*j*_ for *X*_.*j*_ → *M*_.*j*_ → *Y*_*ij*_ (denoted as ie2.2), the indirect effect from *M*_*ij*_ for *X*_.*j*_ → *M*_*ij*_ → *Y*_*ij*_ (denoted as ie2.1), and the total effect (te2) are estimated.

For the level 1 exposure, the average direct effect for *X*_*ij*_ → *Y*_*ij*_ (denoted as de1), the indirect effect from *M*_*ij*_ for *X*_*ij*_ → *M*_*ij*_ → *Y*_*ij*_ (denoted as ie2.1), and the total effect (te1) are estimated.

**Fig 2:**
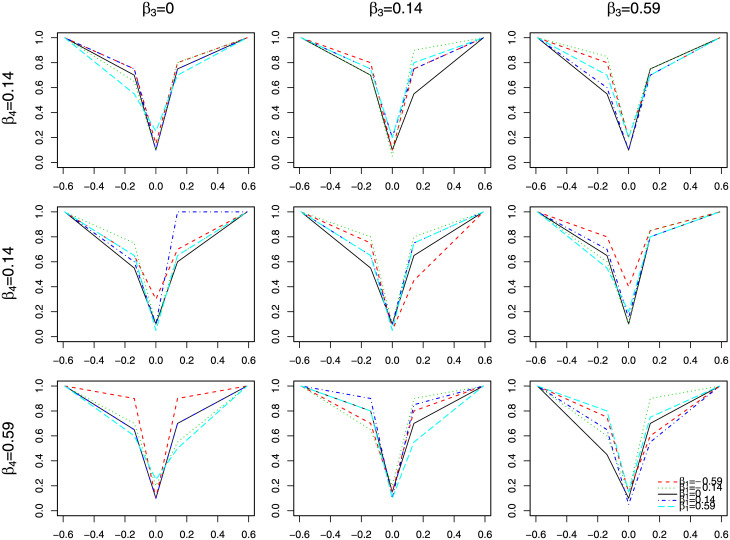
Estimates of level 2 direct effect from simulation 1. The x-axis is the true direct effect, and y-axis is the sensitivity (or 1-specificity when true de is 0). Different color and line type represents different value for *β*_1_. Rows are for *β*_4_ = 0, 0.14 and 0.59, and columns are for *β*_3_ = 0, 0.14 and 0.59 respectively.

Fig 2 shows the sensitivity (or 1-specificity when *β*_2_ = 0) of identifying significant level 2 direct effect, when *β*s change but *n* is fixed at 20 and *v*_1_ at 1. The x-axis is *β*_2_, the true level 2 direct effect. Different color and line type in each plot represents different values of *β*_1_. Each row of the 3 × 3 panel in Fig 2 is a different value for *β*_4_, each column is for a different value of *β*_3_. We found that the sensitivity of correctly identifying important level 2 direct effect depends on the value of other TVEs. This is mainly due to the increased correlations among variables that results in raised variances in the estimation.

**Fig 3:**
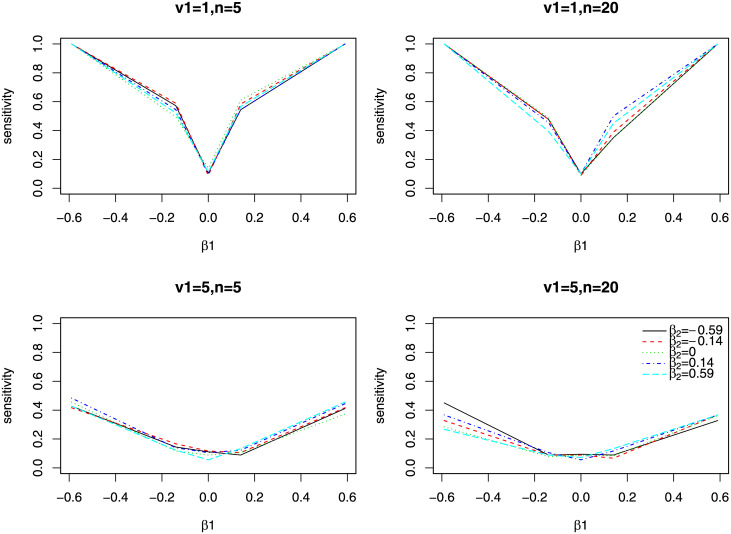
Estimates of level 1 direct effect from Simulation 1. The x-axis is the true direct effect, and y-axis is the sensitivity (or 1-specificity when the true de is 0). Different color and line type represents different value for *β*_2_. Each plot is for a different combination of *n* and *v*_1_.

Fig 3 shows the sensitivity or 1-specificity (when *β*_1_ = 0) of identifying important level 1 direct effect. The sensitivity is averaged over *β*_3_ and *β*_4_ for different values of *β*_1_, *v*_1_ and *n*. We see that the sensitivities reduce a lot when the variances are bigger. However, values of *n* have minimum influence on the sensitivity.

**Fig 4:**
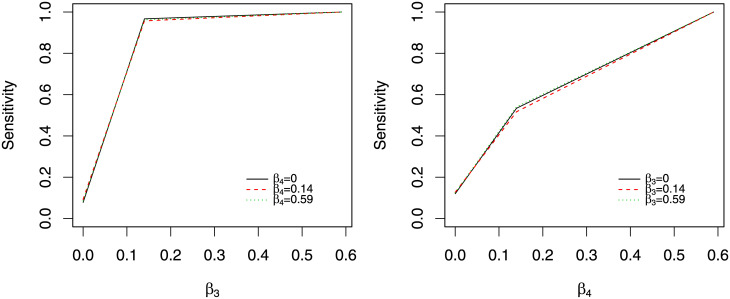
Estimates of level 2 indirect effect from simulation 1. The left panel is the sensitivity and 1-specificity of identifying *M*_*ij*_. The right panel is the sensitivity and 1-specificity of identifying *M*_.*j*_.

**Fig 5:**
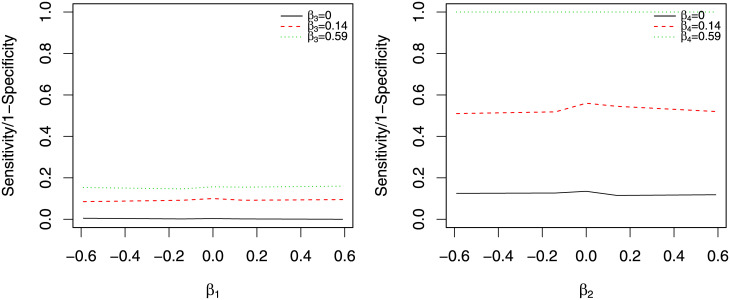
Estimates of indirect effects of *M*_*ij*_ from simulation 1. The left panel is for the level 1 indirect effect and the right panel is for the level 2 indirect effect of *M*_*ij*_.

Finally, Figs 4 and 5 show the sensitivity and specificity of identifying indirect effects. For both Figures, we fix *v*_1_ at 1. Fig 4 shows how the sensitivity of level 2 indirect effects of *M*_*ij*_ and *M*_.*j*_ changes with *β*_3_ and *β*_4_. The left panel is the sensitivity and 1-specificity (when *β*_3_ = 0) of identifying *M*_*ij*_. The level 2 indirect effect of *M*_*ij*_ is proportional to *β*_3_, so the sensitivity increases with *β*_3_. The sensitivity is minimally influenced by the value of *β*_4_. The right panel is the sensitivity and 1-specificity (when *β*_4_ = 0) of identifying *M*_.*j*_. Again, the sensitivity of identifying *M*_.*j*_ is proportional to *β*_4_, but is not systematically influenced by the value of *β*_3_.

Fig 5 shows how the actual direct effect influences the estimation of the indirect effect of *M*_*ij*_. The left panel is for the level 1 indirect effect and the right panel is for the level 2 indirect effect. For both panels, the black solid line is the 1-specificity and the other two lines are the sensitivity. The sensitivity for level 1 indirect effect increases with *β*_3_ and that for level 2 indirect effect increases with *β*_4_. The x-axis is the true level 1 or 2 direct effect for the left and right panel respectively. We see very small influence of the true direct effect on the estimation of the indirect effect.

### 3.2 Simulation 2

The second simulation is to illustrate the use of the *mlma* package with transformations in the variables. In the simulation, there is only one level 2 third-variable, *M*_*ij*_ and the variable is causally proceeded by two exposure variables, *X*_*ij*_ ∼ *N*(0, 0.5) at the first level and *X*_*j*_ ∼ *χ*^2^(*df* = 2) at the second level. In the simulation, there are 30 groups, and 20 observations generated for each group. That is *i* = 1, …, 20 and *j* = 1, …, 30. The data are generated from the following model:

$$\begin{array}{ccc} m_{ij} & = & u_{0j1}+1.2x_{ij}^{2}+1.2x_{ij}+\epsilon_{1ij},\\ y_{ij} & = & u_{0j}+0.98x_{ij}+0.98log\left(x_{.j}\right)+0.98m_{ij}+\epsilon_{ij}, \end{array}$$

where *ϵ*_1*ij*_ ∼ *N*(0, 1), *ϵ*_*ij*_ ∼ *N*(0, 2), *u*_0*j*_ ∼ *N*(0, 0.4) and *u*_*oj*1_ ∼ *N*(0, 0.5) are independently generated as the level 1 and level 2 random errors respectively. For the data generation mechanism, the true level 1 direct effect is 0.98 and the true level 2 direct effect is *de*2(*x*_*j*_) = 0.98/*x*_*j*_. In addition, the true level 1 indirect effect for *M* at *x*_*ij*_ is 1.2 × 0.98 × *x*_*ij*_ and the true level 2 indirect effect is 1.2 × 0.98 = 1.176, where the former depends on the value of *x*_*ij*_ representing a nonlinear relationship between *M* and *X*_*ij*_ and the latter represents a strong (1.176) constant indirect effect.

**Fig 6:**
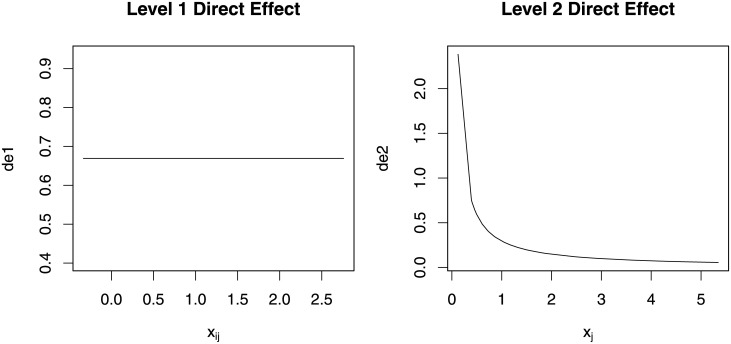
Estimates of direct effects of at both levels for simulation 2.

Fig 6 shows the estimated direct effects at both levels from the simulated data. We see that the estimated direct effect correctly delineates the nonlinear relationship among variables.

**Fig 7:**
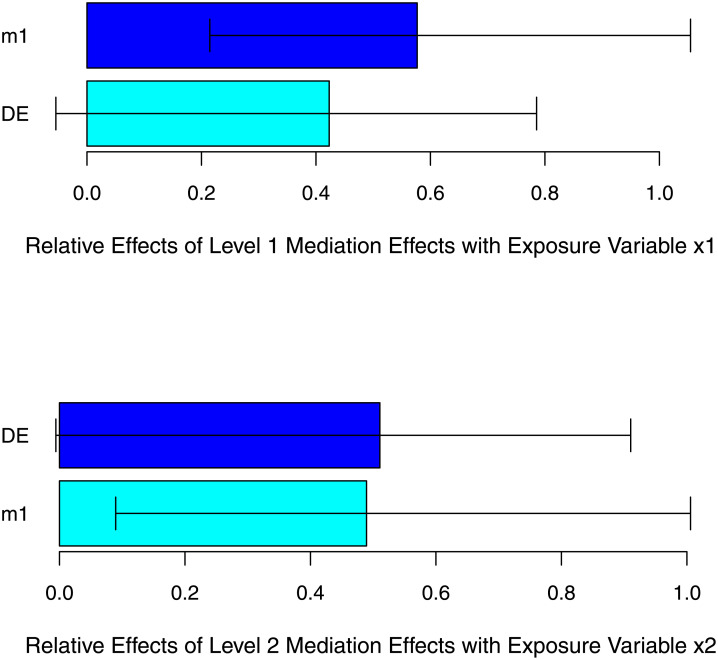
Relative effect at different exposure variables for simulation 2.

The *mlma* package provides a *plot* function to show relationships among variables. Fig 7 shows the results of the *plot* function without specifying a specific third-variable. The Figure shows the relative effect (defined as the TVE divided by the total effect) at each exposure variable.

**Fig 8:**
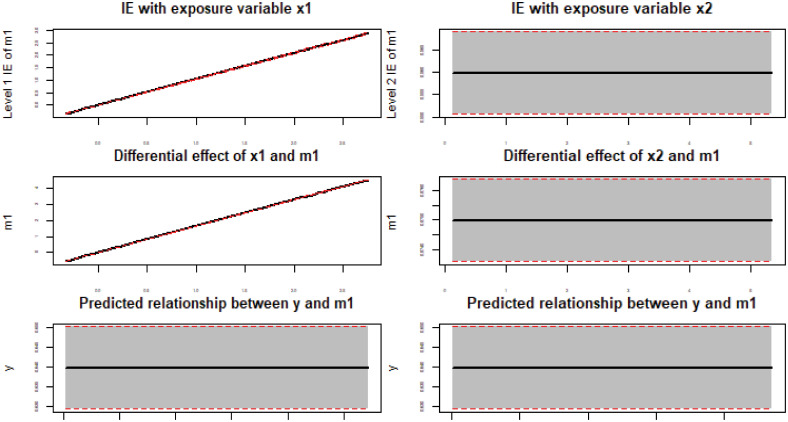
Indirect effect of *M* at different exposure variables for simulation 2.

Fig 8 shows results of the *plot* function with the third-variable *M*. The left panel is the effect of *M* with the level 1 exposure denoted as *x*1 and the right panel is for the level 2 exposure which is denoted as *x*2. The first row of Fig 8 gives the estimated indirect effect with confidence intervals. The second row shows the changing rate of *M* with the exposure variables and the third row gives the changing rate of the outcome with the third-variable *M*. Again, the figures correctly describe relationships among variables.

The codes for simulating data set and transforming variables in the third-variable analysis are available in the S1 File.

## 4 Explore the racial disparity in obesity

Finally, we implement the method in a real data example: to explore the racial disparity in body mass index (BMI). In a previous research, we found that on average, non-Hispanic blacks have a higher BMI and higher rate of obesity compared with non-Hispanic whites [19, 22] using the 2003-2006 National Health and Nutrition Examination Survey (NHANES). We are interested to see how the disparities can be explained by both individual and environmental factors. Environmental risk factors are generated at the census tract level, which include both food environments and physical activity environments. Individual level variables include age, gender, smoking status, etc. Readers are referred to [19, 22] for more details about the variables.

In this demonstration, we try to use risk factors to explain the racial disparity in BMI. We first use multiple additive regression trees to describe the relationships between BMI and all risk factors, and then we performed a data transformation on the risk factors so that the transformed variables have a roughly linear relationship with BMI. For the individual level risk factors, we did the following transformations:

The natural cubic spline bases for age with degrees of freedom of 4.
Truncate the physical activity measurement to two parts: smaller than 2.1 and larger than 2.1 since we see a change point at 2.1 when depicting the relationship between physical activity and BMI.

We also use the natural cubic spline basis with different degrees of freedom on some of the environmental factors. Please refer to the S1 File to see how we make the transformations. The individual level exposure variable is the race (black (0) and white (1)). The census tract level exposure is the proportion of whites in the census tract. Table 1 shows the estimated third variable effects at both the individual and census tract levels. Note that since high level variable (e.g. census tract level) can influence the lower level (individual level) variable but not the reverse, all level 1 third-variables have both individual and census tract level effects, while all level 2 third-variables have only census tract level effects.

**Table 1:** Inferences on third-variable effects of risk factors in explaining racial disparities in BMI.

	Individual Level	Census Tract Level
Total Effect	-1.94(-2.39,-1.07)	-0.09(-1.03,0.98)
Race (direct effect)	-1.46(-2.05,-0.72)	-0.08(-0.86,0.72)
Age	-0.30(-0.28,-0.17)	-0.01(-0.030,0.01)
Foreign Born	-0.11(-0.14,-0.07)	0.02(-0.01,0.04)
Smoker	0.06(0.03,0.09)	0.05(-0.02,0.11)
Sex	-.004(-.01,-.003)	-0.01(-.03,0.01)
Physical Activity	-0.08(-.01,-0.03)	-0.21(-0.39,-0.06)
Elevation	-	-0.04(-0.43,0.60)
Street Density	-	-0.07(-.28,0.18)
Connected Node Ratio	-	0.00(-0.00,0.00)
Intersection Density	-	0.25(-0.15,0.51)

**Fig 9:**
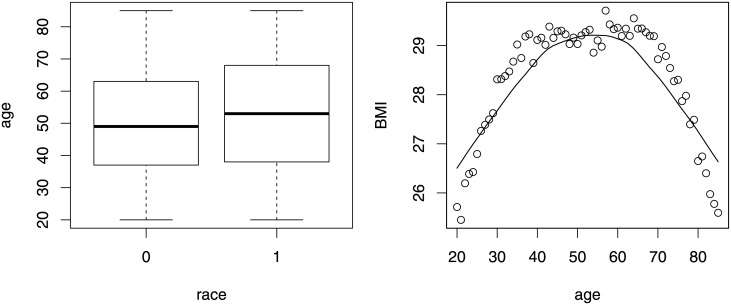
Indirect effect of age at the individual level.

We found that at the individual level, on average, the BMI among Whites is 1.94 (TE) lower than that for Blacks. Individual level factors can partially explain the racial difference. For example, age explains about 15%(−0.30 *dividedby* −1.94) of the difference. Fig 9 shows that a larger proportion of Blacks (*x* = 0) are at the middle age (late 30s to early 60s), compared with Whits. In addition, middle age is related with the highest BMI. The right panel of Fig 9 shows the relationship between age and BMI, which is not linear. BMI increases with age, peaked at the middle age and then declines. By the transformation in third-variable analysis, we can catch the nonlinear relationship between age and BMI.

On the other hand, the racial difference in BMI at the census tract level is not significant. The total effect is −0.9 with a 95% confidence interval that includes 0. In the study, no environmental risk factors that significantly influences the racial disparity in BMI is found. However, in the analysis, multilevel model is required to control the dependencies of subjects who come from the same residential environments.

## 5 Discussion and future work

In this paper, we extend the definitions of TVEs proposed by [7] to multilevel data settings, based on which the direct and indirect effects can be differentiated from different levels. In addition, indirect effects from different third-variables can be separated. Multilevel additive models are adapted to model the relationships among variables to account for the hierarchical data structure. Data transformation is allowed to represent potential non-linear relationship. We also create an R package, *mlma*, that implements the proposed method for multilevel third-variable analysis. Using the package, TVEs on average as well as at different values of the exposure(s) can be estimated. Simulations showed that the proposed method and package can accurately estimate TVEs at different levels. We implement the method to explore the racial disparity in BMI accounting for both environmental and individual-level risk factors.

There are some limitations in the current method. One is that we have to know how to transform the variables so that the multilevel additive linear models can accurately catch the relationships among variables. Also, the transformation functions have to be differentiable to calculate the indirect effects. Alternative, we can use the multilevel smoothing splines [30], to analyze the multilevel relationship. In such case, there is no need to know the transformation forms beforehand and the bases for smoothing splines are differentiable. Another limitation of the package of the current version is that the research is confined to deal with data of at most two levels. Extension to more levels is more complicated due to the conditions of TVEs such that the predictor should have equal or higher level than the third-variables, which in turn should have higher or equal levels than the outcome. Furthermore, when the dataset has more levels, TVEs from different levels should be estimated separately. A future work is to develop a more flexible algorithm for general models of potentially more levels.

## Supporting information

S1 File Proofs and R codes. (PDF)
