# Supplementary material for: Third-variable effect analysis with multilevel additive models
Source: PLoS One. 2020 Oct 23;15(10):e0241072. doi: 10.1371/journal.pone.0241072 (PMC7584256; doi:10.1371/journal.pone.0241072)
Supplement: S1 File — (PDF) [file pone.0241072.s001.pdf]

Third-Variable Effect Analysis with Multilevel Additive Models

Supplemental Material

August 17, 2020

1 Proof for Theorems 1 and 2

1.1 Proof for Theorem 1

Proof. For the third-variable effects at the first level, by the multilevel additive models described in Section 2.3, we have that

$$E(Y_{ij}) = u_{0j} + \sum_{e=1}^{E_1} {\boldsymbol{\beta}_{1e}}^T \mathbf{f}_{1e}(X_{ije}) + \sum_{e=1}^{E_2} {\boldsymbol{\beta}_{2e}}^T \mathbf{f}_{2e}(X_{.je}) + \sum_{k=1}^{K} {\boldsymbol{\beta}_{3k}}^T \mathbf{f}_{3k}(M_{ijk}) + \sum_{l=1}^{L} {\boldsymbol{\beta}_{4l}}^T \mathbf{f}_{4l}(M_{.jl}).$$

In addition, we have that for $k = 1, \ldots, K$,

$$g_{1k}(E(M_{ijk})) = u_{0jk} + \sum_{e=1}^{E_1} {\boldsymbol{\alpha}_{1ke1}}^T \mathbf{f}_{1ke1}(X_{ije}) + \sum_{e=1}^{E_2} {\boldsymbol{\alpha}_{2ke1}}^T \mathbf{f}_{2ke1}(X_{.je}).$$

Denoting that $\mu_{ijk} = E(M_{ijk})$, by Equation (1) in Definition 1, we have

$$\begin{aligned}
TE_1(x_{ije}) &= \left.\frac{\partial E(Y_{ije})}{\partial X_{ije}}\right|_{X_{ije}=x_{ije}} \\
&= \left[\frac{\partial \boldsymbol{\beta}_{1e}^T \mathbf{f}_{1e}(X_{ije})}{\partial X_{ije}} + \sum_{k=1}^{K} \frac{\partial \boldsymbol{\beta}_{3k}^T \mathbf{f}_{3k}(M_{ijk})}{\partial M_{ijk}} \cdot \frac{\partial M_{ijk}}{\partial X_{ije}}\right]_{X_{ije}=x_{ije}} \\
&= {\boldsymbol{\beta}_{1e}}^T \left.\frac{\partial \mathbf{f}_{1e}(X_{ije})}{\partial X_{ije}}\right|_{X_{ije}=x_{ije}} \\
&\quad + \sum_{k=1}^{K} \left[\boldsymbol{\beta}_{3k}^T \left.\frac{\partial \mathbf{f}_{3k}(M_{ijk})}{\partial M_{ijk}}\right|_{M_{ijk}=m_{ijk}} \cdot \boldsymbol{\alpha}_{1ke1}^T \left.\frac{\partial \mathbf{f}_{1ke1}(X_{ije})}{\partial X_{ije}}\right|_{X_{ije}=x_{ije}} \cdot \left.\frac{\partial g_{1k}^{-1}(M_{ijk})}{\partial M_{ijk}}\right|_{M_{ijk}=\mu_{ijk}}\right] \\
&= DE_1(x_{ije}) + \sum_{k=1}^{K} IE_{1,k}(x_{ije})
\end{aligned}$$

□

1.2 Proof for Theorem 2

Proof. For the third-variable effects at the second level, by the multilevel additive models described in Section 2.3, we have that

$$E(Y_{ij}) = u_{0j} + \sum_{e=1}^{E_1} \boldsymbol{\beta}_{1e}{}^T \mathbf{f}_{1e}(X_{ije}) + \sum_{e=1}^{E_2} \boldsymbol{\beta}_{2e}{}^T \mathbf{f}_{2e}(X_{.je}) + \sum_{k=1}^{K} \boldsymbol{\beta}_{3k}{}^T \mathbf{f}_{3k}(M_{ijk}) + \sum_{l=1}^{L} \boldsymbol{\beta}_{4l}{}^T \mathbf{f}_{4l}(M_{.jl}).$$

In addition, we have that for $k = 1, \ldots, K$,

$$g_{1k}(E(M_{ijk})) = u_{0jk} + \sum_{e=1}^{E_1} \boldsymbol{\alpha}_{1ke1}{}^T \mathbf{f}_{1ke1}(X_{ije}) + \sum_{e=1}^{E_2} \boldsymbol{\alpha}_{2ke1}{}^T \mathbf{f}_{2ke1}(X_{.je});$$

and for $l = 1, \ldots, L$,

$$g_{.l}(E(M_{.jl})) = \alpha_{0l} + \sum_{e=1}^{E_2} \boldsymbol{\alpha}_{2le}{}^T \mathbf{f}_{2le}(X_{.je}).$$

Denoting that $\mu_{ijk} = E(M_{ijk})$ and $\mu_{.jk} = E(M_{.jk})$, by Equation (4) in Definition 2, we have

$$\begin{aligned}
TE_2(x_{.je}) &= \left.\frac{\partial E(Y_{ije})}{\partial X_{.je}}\right|_{X_{.je}=x_{.je}} \\
&= \left[\frac{\partial \boldsymbol{\beta}_{2e}^T \mathbf{f}_{2e}(X_{.je})}{\partial X_{.je}} + \sum_{k=1}^{K} \frac{\partial \boldsymbol{\beta}_{3k}^T \mathbf{f}_{3k}(M_{ijk})}{\partial M_{ijk}} \cdot \frac{\partial M_{ijk}}{\partial X_{.je}} + \sum_{l=1}^{L} \frac{\partial \boldsymbol{\beta}_{4l}^T \mathbf{f}_{4l}(M_{.jl})}{\partial M_{.jl}} \cdot \frac{\partial M_{.jl}}{\partial X_{.je}}\right]_{X_{.je}=x_{.je}} \\
&= \boldsymbol{\beta}_{2e}{}^T \left.\frac{\partial \mathbf{f}_{2e}(X_{.je})}{\partial X_{.je}}\right|_{X_{.je}=x_{.je}} \\
&\quad + \sum_{k=1}^{K} \left[\boldsymbol{\beta}_{3k}^T E\left[\left.\frac{\partial \mathbf{f}_{3k}(M_{ijk})}{\partial M_{ijk}}\right|_{M_{ijk}=m_{ijk}}\right] \cdot \boldsymbol{\alpha}_{2ke1}^T \left.\frac{\partial \mathbf{f}_{2ke1}(X_{.je})}{\partial X_{.je}}\right|_{X_{.je}=x_{.je}} \cdot \left.\frac{\partial g_{1k}^{-1}(M_{ijk})}{\partial M_{ijk}}\right|_{M_{ijk}=\mu_{ijk}}\right] \\
&\quad + \sum_{l=1}^{L} \left[\boldsymbol{\beta}_{4l}^T \left.\frac{\partial \mathbf{f}_{4l}(M_{.jl})}{\partial M_{.jl}}\right|_{M_{.jl}=m_{.jl}} \cdot \boldsymbol{\alpha}_{2le}^T \left.\frac{\partial \mathbf{f}_{2le}(X_{.je})}{\partial X_{.je}}\right|_{X_{.je}=x_{.je}} \cdot \left.\frac{\partial g_{.l}^{-1}(M_{.jl})}{\partial M_{.jl}}\right|_{M_{.jl}=\mu_{.jl}}\right] \\
&= DE_2(x_{.je}) + \sum_{k=1}^{K} IE_{21,k}(x_{.je}) + \sum_{l=1}^{L} IE_{22,l}(x_{.je})
\end{aligned}$$

□

2 R codes for Simulations and Real Example

2.1 Simulation 1

Data generation and third-variable analysis

```
library(mlma)
set.seed(1)
alpha_211<-0.8
alpha_1111<-0.8
alpha_2111<-0.8

effect1<-NULL
effect2<-NULL
parameters<-NULL

set.seed(1)
for (n in c(5,20))
 for (beta_1 in c(-0.59, -0.14, 0, 0.14, 0.59))
  for (beta_2 in c(-0.59, -0.14, 0, 0.14, 0.59))
   for (beta_3 in c(0, 0.14, 0.59))
    for (beta_4 in c(0, 0.14, 0.59))
     for (v1 in c(1, 5))
      for (k in 1:20)
{J<-600/n
 level=rep(1:J,each=n)
 v2=v1/5
 x1<-rbinom(600,1,0.5) #binary level 1 exposure, xij
 x2<-rep(rnorm(J),each=n) #continuous level 2 exposure
 u1<-rep(rnorm(J,0,0.5),each=n) #level 2 variance for mij
 e1<-rnorm(n*J)  #level 1 variance for mij
 m2<-rep(rbinom(J,1,exp(alpha_211*unique(x2))/(1+exp(alpha_211*unique(x2)))),
        each=n)    #level 2 binary mediator
 m1<-u1+alpha_1111*x1+alpha_2111*x2+e1 #level 1 continuous mediator
 u0<-rep(rnorm(J,0,v2),each=n)
 e0<-rnorm(n*J,0,v1)
 y<-u0+beta_1*x1+beta_2*x2+beta_3*m1+beta_4*m2+e0
```

```
 example1<-data.org(x=cbind(x1=x1,x2=x2), m=cbind(m1=m1,m2=m2),
                   level=level)
 result2<-summary(boot.mlma(y=y, data1=example1,boot=50,intercept=F,echo=F))
 effect1<-rbind(effect1,result2$effect1)
 effect2<-rbind(effect2,result2$effect2)
 parameters<-rbind(parameters, c(n, beta_1, beta_2, beta_3, beta_4, v1))
}
```

2.2 Simulation 2

```
set.seed(3)
x1<-rnorm(600,1,0.5) #continuous level 1 exposure, xij
x2<-rep(rchisq(30, df=2),each=20) #continuous level 2 exposure
u1<-rep(rnorm(30,0,0.5),each=20) #level 2 variance for mij
e1<-rnorm(600)  #level 1 variance for mij
m1<-u1+0.98*x1^2+0.98*x2+e1 #level 1 continuous mediator
u0<-rep(rnorm(30,0,0.4),each=20)
e0<-rnorm(600,0,2)
y<-u0+1.2*x1+1.2*log(x2)+1.2*m1+e0
example2<-data.org(x=cbind(x1=x1,x2=x2), m=data.frame(m1=m1),
                   f01y=list(2,c("log(x)")),f10km=list(matrix(c(1,1),1),"x^2"),
                   level=level)
result.2<-boot.mlma(y=y, data1=example2,boot=100,intercept=F)
```

2.3 The Real Example

```
x<-dataset4[,"black"] # the vector of predictor
m4<-dataset4[,c("Elevation", "POP00_SQMI", "mvper","male",
                "povcat", "foreign", "csmoker", "age", "hisp","strden00",
                "unhpopChi","cnr01","Bars05_POP00","intden00","Con_POP00_Chi")]
y4<-dataset4[,"bmxbmi"]   # outcome is bmi (continuous)

m4.2<-m4[,c("Elevation", "PhysicalActivity","Male",
```

```
            "ForeignBorn", "Smoker", "age", "StreetDensity",
            "ConnectedNodeCat","IntersectionDensity")]

data4.2<-data.org(x=x, levelx=1, m=m4.2, xref=1,
                  l1=c("PhysicalActivity","age"),
                  l2=c("Elevation", "StreetDensity",
                       "IntersectionDensity"),
                  c1=c("Male", "ForeignBorn", "Smoker"),
                  c1r=c(1, 1, 1),  #check the reference group
                  c2=c("ConnectedNodeCat"),
                  c2r=c(0),
                  f02ky=list(c("Elevation", "StreetDensity","IntersectionDensity"),
                             c("ns(x,df=5)"),
                             c("ns(x,df=4)"),
                             c("ns(x,df=3)")),
                  f20ky=list(c("age","PhysicalActivity"),
           c("ns(x,df=4)"),  #the transformation for age and PA
           c("ifelse(x<2.1,x,2.1)","ifelse(x>2.1,x-2.1,0)")),
                  level=fips)

mlma4.2<-mlma(y=y4,  data1=data4.2,
              joint=list(c(2),c("StreetDensity","IntersectionDensity","ConnectedNodeCat")))

mlma4.b<-boot.mlma(y=y4,  data1=data4.2, boot=500,
              joint=list(c(2),c("StreetDensity","IntersectionDensity","ConnectedNodeCat")))

summary(mlma4.b)
plot(mlma4.b)
plot(mlma4.b,var="age")
plot(mlma4.2,var="age")
```
